# Real-world effectiveness of third-line cabazitaxel in patients with metastatic castration-resistant prostate cancer: CARD-like analysis of data from a post-marketing surveillance in Japan

**DOI:** 10.1186/s12885-023-10998-w

**Published:** 2023-06-13

**Authors:** Hideyasu Matsuyama, Nobuaki Matsubara, Hirotaka Kazama, Takeshi Seto, Yoshinori Sunaga, Kazuhiro Suzuki

**Affiliations:** 1 Department of Urology, Graduate School of Medicine, Yamaguchi University, Yamaguchi, Japan; 2 Present Address: Department of Urology, JA Yamaguchi Kouseiren Nagato General Hospital, Yamaguchi, Japan; 3 Department of Medical Oncology, National Cancer Center Hospital East, Chiba, Japan; 4 Speciality Care Oncology Medical, Sanofi, Tokyo, Japan; 5 Medical Affairs, Sanofi, Tokyo, Japan; 6 Department of Urology, Gunma University Graduate School of Medicine, Gunma, Japan

**Keywords:** Metastatic castration-resistant prostate cancer, Androgen receptor-axis-targeted therapy, Cabazitaxel, Post-marketing surveillance, Real-world data, Sequential treatment, Cross-resistance, Effectiveness, Japanese, Time to treatment failure

## Abstract

**Background:**

The CARD trial was conducted in patients with metastatic castration-resistant prostate cancer (mCRPC) who had received docetaxel and experienced disease progression within 1 year on an androgen receptor-axis-targeted therapy (ARAT). Subsequent treatment with cabazitaxel had improved clinical outcomes compared with an alternative ARAT. This study aims to confirm the effectiveness of cabazitaxel in real-world patients in Japan and compare their characteristics with those of patients from the CARD trial.

**Methods:**

This was a post-hoc analysis of a nationwide post-marketing surveillance registering all patients who were prescribed cabazitaxel in Japan between September 2014 and June 2015. Included patients had received docetaxel and ≤ 1 year of an ARAT (abiraterone or enzalutamide) prior to receiving cabazitaxel or an alternative ARAT, as their third-line therapy. The primary effectiveness endpoint was the time to treatment failure (TTF) of the third-line therapy. Patients were matched (1:1) from the cabazitaxel and second ARAT arms based on propensity score (PS).

**Results:**

Of the 535 patients analysed, 247 received cabazitaxel and 288 the alternative ARAT as their third-line therapy, of which, 91.3% (*n* = 263/288) received abiraterone and 8.7% (*n* = 25/288) received enzalutamide as their second third-line ARAT. Patients in the cabazitaxel and second ARAT arms had TNM classification of M1 or MX in 73.3% and 68.1%, Gleason score of 8–10 in 78.5% and 79.2% and mean (standard deviation) serum PSA levels of 483 (1370) and 594 (1241) ng/mL, respectively. Initial cabazitaxel dose was ≤ 20 mg/m^2^ in 61.9% (*n* = 153/247) of the patients in the cabazitaxel arm. The median TTF (95% confidence interval [CI]) of the third-line therapy was 109 (94–128) days for cabazitaxel and 58 (57–66) days for the second ARAT, with a hazard ratio (95% CI) of 0.339 (0.279–0.413) favouring cabazitaxel. Similar results were obtained after PS-matching, with a hazard ratio (95% CI) of 0.323 (95% CI 0.258–0.402) favouring cabazitaxel.

**Conclusions:**

Consistent with the CARD trial, cabazitaxel demonstrated superior effectiveness over a second alternative ARAT in a real-world patient population in Japan, despite the population having more advanced disease status and a lower dose of cabazitaxel being more frequently administered, than in the CARD trial.

**Supplementary Information:**

The online version contains supplementary material available at 10.1186/s12885-023-10998-w.

## Background

Prostate cancer (PC) is one of the most common types of cancer among men; age-standardized rate of PC worldwide was 30.7 per 100,000 person-years in 2020 [1]. Despite favourable response to initial treatment, many men with PC then experience progression due to treatment resistance [2]. Metastatic disease is the leading cause of death from PC, with an overall survival (OS) of 16–18 months on average after disease progression [2, 3].

Androgen deprivation therapy (ADT) with castration remains the standard initial treatment for PC [2] and docetaxel chemotherapy has been the standard first-line treatment for metastatic PC since 2004 [2, 4]. Androgen receptor-axis-targeted therapies (ARATs)^1^, such as abiraterone or enzalutamide, are newer ADTs that are now commonly used for first-line treatment of metastatic castration-sensitive PC (mCSPC) as well as metastatic castration-resistant PC (mCRPC) [5, 6]. Subsequent treatment options for mCRPC have expanded beyond the first line to include either an alternative ARAT or cytotoxic taxane-based chemotherapy, such as cabazitaxel [7]. With increasing use of ARATs and taxanes in earlier lines of treatment, cross-resistance between therapies due to shared mechanisms of resistance [8] has emerged as an impediment in treatment of mCRPC at later lines. Sequential use of ARATs for the treatment of mCRPC is frequent in clinical practice [9, 10], despite guidance in Europe and the US cautioning against such use [11, 12].

Cabazitaxel was approved for treatment of CRPC in patients who were previously treated with docetaxel and is recommended at 20 or 25 mg/m^2^ in combination with oral prednisolone [13–15]. The CARD trial was a randomised clinical trial of mCRPC patients who have previously received docetaxel and experienced disease progression within 1 year on an ARAT, who then received either 25 mg/m^2^ cabazitaxel or an alternative ARAT [16]. The CARD trial reported significantly improved imaging-based progression-free survival (PFS) and OS in patients receiving cabazitaxel compared with those receiving an alternative ARAT [16].

Subsequently, de Wit et al. have applied these findings to a real-world data analysis of a CARD-like cohort using a global oncology database and found that the results from the CARD trial reflect the characteristics of patients in real-world clinical practice [9]. Given that the CARD trial suggested that cabazitaxel is more effective than an alternative ARAT, this study aims to retrospectively confirm its effectiveness in real-world patients and whether the results from de Wit’s real-world analysis [9] also apply to Japanese patients. This study also aims to identify any patterns of cross-resistance to treatment arising from sequential use of ARATs or taxane-based chemotherapies in a real-world population, by applying the analysis from the CARD trial to a real-world dataset from a Japanese post-marketing surveillance (PMS) of cabazitaxel [17].

## Methods

### Study design and data source

This study reports a post-hoc analysis of data from a nationwide all-case PMS registering all patients who were prescribed cabazitaxel in Japan between September 2014 and June 2015 [17]. During the PMS, case report forms were completed by the investigators for up to 1 year. Collected data included patient demographics, disease characteristics, cabazitaxel exposure, prior and concomitant therapies, prostate-specific antigen (PSA) levels, and last survival dates.

### Objectives and patient cohorts

The objectives of this study were to: 1) investigate whether the patient characteristics of the CARD trial cohort reflect a real-world patient population in Japan, 2) compare the effectiveness of cabazitaxel with abiraterone or enzalutamide at third-line treatment in patients who had received docetaxel and ≤ 1 year of an alternative ARAT in prior lines of treatment, and 3) assess cross-resistance to treatment arising from sequential use of ARATs (abiraterone and enzalutamide) or taxane-based chemotherapies (docetaxel and cabazitaxel). 

To identify a “CARD-like” patient cohort within patients registered in the Japanese PMS for cabazitaxel [17], we searched for those who had received the following treatments prior to their third-line therapy: ≥ 1 cycle of docetaxel and ≤ 1 year of an ARAT (abiraterone or enzalutamide) either before or after docetaxel. Patients were excluded from the analysis if they stayed on their first ARAT for > 1 year. Patients were then grouped according to the therapy received at the third line: cabazitaxel or the second alternative ARAT.

In a subanalysis, patients were matched (1:1) from the cabazitaxel and second ARAT arms based on propensity score (PS) to control for possible confounding patient characteristics.

### Effectiveness endpoints

The effectiveness endpoint was the time to treatment failure (TTF) of the third line therapy, which was assessed for up to 1 year. TTF of cabazitaxel was defined as the time from the first day of treatment to 30 days after the last day of treatment or death from any cause, whichever occurred first. TTF of the second ARAT was defined as the time from the first day to the last day of treatment. Patients were assessed for survival up to 1 year after starting cabazitaxel treatment. Survival of patients during the second ARAT at third line was inestimable in this study (included patients had to survive and receive cabazitaxel in subsequent lines of treatment).

### Statistical methods

Patient characteristics were collected at the start of cabazitaxel treatment and summarized by descriptive statistics. Continuous variables were described by their mean, median, standard deviation (SD), and range and categorical variables were described by the number of patients and proportion (%).

For PS matching, the a priori probability of a patient being in either treatment group (cabazitaxel or second ARAT) was estimated using a logistic regression model constructed based on patient characteristics. Baseline characteristics before the third-line treatment were unknown for patients in the second ARAT arm because they received cabazitaxel as a fourth- or fifth-line treatment, therefore, covariates, which were deemed as unlikely to change systematically over treatment lines were used in the logistic model: age, body surface area, Gleason score, curative intent focal therapy, number of docetaxel treatment cycles, and reason for discontinuation of docetaxel. A nearest neighbour method without replacement (calliper width: 0.2 SD) was used to match the patients 1:1 from each treatment arm (i.e., the PS-matched CARD-like cohort).

Kaplan–Meier (KM) curves were used to estimate the medians of TTF and OS with 95% confidence intervals (CIs). The effect of treatment on TTF was assessed by the log-rank test at the significance level of 0.05 (two-sided). A Cox proportional hazards model was used to estimate the hazard ratio (HR) with 95% CI of the effect of cabazitaxel and second ARAT on treatment failure in the unmatched and PS-matched CARD-like cohorts. Additionally, a multivariate analysis using the Cox proportional hazards model incorporating the same 6 covariates used for PS matching was performed in the unmatched CARD-like cohort. Software SAS (version 9.4) from SAS Institute (Cary, NC, USA) was used for the statistical analyses.

## Results

### A CARD-like patient cohort within a Japanese PMS cohort

**Fig. 1:**
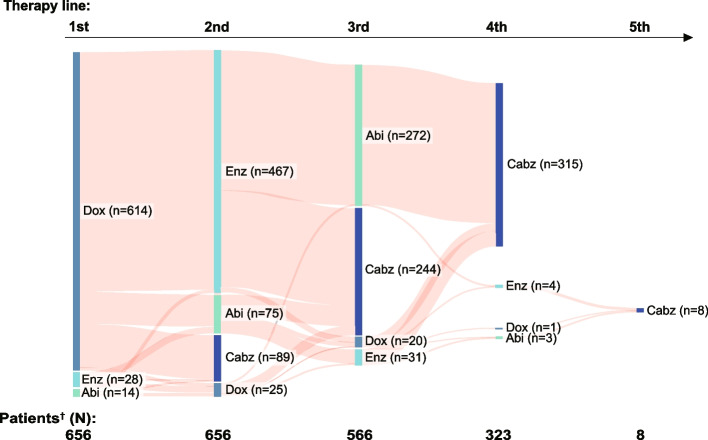
Sanky diagram of treatment patterns across lines of therapy in Japanese patients with mCRPC who received cabazitaxel treatment (*n* = 660). ^†^Prior treatments to cabazitaxel were unknown for four patients (*n* = 4). *Abi* abiraterone; *Cabz* cabazitaxel; *Doc* docetaxel; *Enz* enzalutamide; *mCRPC* metastatic castration-resistant prostate cancer

**Fig. 2:**
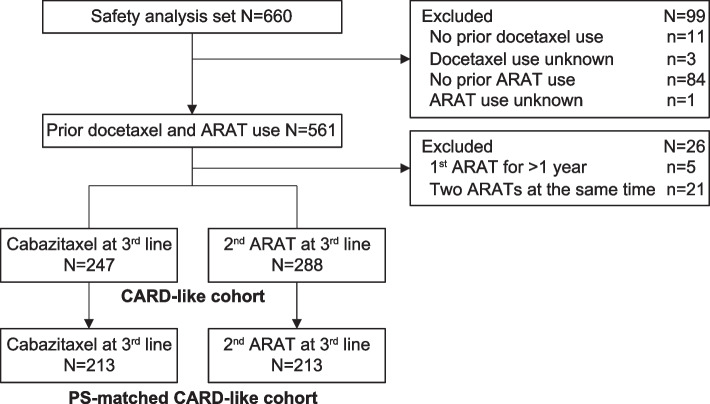
Patient disposition. Patients who had not received cabazitaxel by their second-line treatment were grouped according to the therapy received at the third line (cabazitaxel or an ARAT [abiraterone or enzalutamide]). *ARAT* androgen receptor axis-targeted agent; *PS* propensity score

Figure 1 shows the treatment pattern across lines of therapy in all 660 patients with mCRPC who were registered in a Japanese PMS for cabazitaxel [17]. Of 561 patients who had known docetaxel and ARAT use (abiraterone or enzalutamide) prior to their third-line treatment, 26 were excluded because they received their first ARAT for > 1 year (*n* = 5) or received two ARATs concomitantly (*n* = 21; Fig. 2). Of the remaining 535 patients (i.e., CARD-like cohort) who switched to another therapy within a year after the first ARAT treatment, 247 received cabazitaxel and 288 the alternative ARAT (i.e., second ARAT arm) as their third-line therapy. Of the 288 patients in the second ARAT arm, 263 (91.3%) received abiraterone and 25 (8.7%) received enzalutamide as their second ARAT. After applying PS matching between the cabazitaxel and second ARAT arms, each group comprised 213 patients (Fig. 2).

Patient characteristics were balanced between the cabazitaxel and second ARAT arms in both unmatched and PS-matched CARD-like cohorts (Table 1), despite the characteristics being collected at the start of third line for the cabazitaxel arm and fourth or fifth line for the second ARAT arm (when patients received their first cabazitaxel treatment). In the unmatched cohort, the median age of the patients was 71.0 years old for both the cabazitaxel and second ARAT arms, with 28.7% and 26.0% of the patients being ≥ 75 years old, respectively. Patients in the cabazitaxel and second ARAT arms had TNM classification of M1 (evidence of distant metastasis) or MX (metastasis cannot be measured) in 73.3% and 68.1%, Gleason score of 8–10 in 78.5% and 79.2%, and mean (SD) serum PSA levels of 483 (1370) ng/mL and 594 (1241) ng/mL, respectively. Patients in both arms received a median of 10 cycles of docetaxel treatment (range: cabazitaxel arm, 1–143 cycles; second ARAT arm, 1–83 cycles). Initial cabazitaxel dose was ≤ 20 mg/m^2^ in 61.9% (*n* = 153/247) of patients in the cabazitaxel arm. Exposure to cabazitaxel, PSA response, and frequency of treatment discontinuation due to adverse drug reactions (ADRs), by initial cabazitaxel dose (≤ 20 or > 20 mg/m2) in the cabazitaxel arm are shown in Table S1 (Additional file 1). Details of ADRs reported in patients receiving cabazitaxel are provided in Table S2 (Additional file 1).

**Table 1:** Patient characteristics

**Characteristic**^**a**^	**CARD-like cohort (*N* = 535)**		**PS-matched**^**b**^** CARD-like cohort (*N* = 426)**	
	**Cabazitaxel at 3**^**rd**^** line** **(*N* = 247)**	**2**^**nd**^** ARAT**^**c**^** at 3**^**rd**^** line** **(*N* = 288)**	**Cabazitaxel at 3**^**rd**^** line** **(*N* = 213)**	**2**^**nd**^** ARAT**^**c**^** at 3**^**rd**^** line** **(*N* = 213)**
Age, years				
Median (range)	71.0 (46–91)	71.0 (43–89)	70.0 (46–91)	71.0 (43–89)
≥ 75, n (%)	71 (28.7)	75 (26.0)	54 (25.4)	57 (26.8)
Body surface area, m^2^				
Median (range)	1.66 (1.29–2.13)	1.65 (1.26–2.06)	1.66 (1.29–2.13)	1.65 (1.30–2.06)
Duration of disease, years				
Median (range)	4.06 (0.5–19.8)	4.34 (1.0–17.5)	4.07 (0.5–19.8)	4.18 (1.0–17.5)
Gleason score, n (%)				
2–7	42 (17.0)	41 (14.2)	37 (17.4)	31 (14.6)
8–10	194 (78.5)	228 (79.2)	176 (82.6)	182 (85.5)
TNM classification, n (%)				
T1 + T2	43 (17.4)	51 (17.7)	38 (17.8)	38 (17.8)
T3 + T4 + TX	200 (81.0)	233 (80.9)	171 (80.3)	171 (80.3)
N0	113 (45.8)	136 (47.2)	101 (47.4)	101 (47.4)
N1 + NX	134 (54.3)	149 (51.7)	112 (52.6)	109 (51.2)
M0	66 (26.7)	91 (31.6)	60 (28.2)	70 (32.9)
M1 + MX	181 (73.3)	196 (68.1)	153 (71.8)	142 (66.7)
ECOG PS score, n (%)				
0	145 (58.7)	187 (64.9)	127 (59.6)	139 (65.3)
≥ 1	102 (41.3)	100 (34.7)	86 (40.4)	73 (34.3)
PSA, ng/mL				
Median (range)	139 (0–16,697)	228 (0–10,027)	150 (0–16,697)	195 (0–10,027)
Neutrophil count, per mm^3^				
Median (range)	4430 (81–15,380)	4500 (239–10,290)	4500 (81–9983)	4452 (239–10,220)
Medical history, n (%)	76 (30.8)	91 (31.6)	70 (32.9)	73 (34.3)
Complications, n (%)	114 (46.2)	107 (37.2)	97 (45.5)	77 (36.2)
Prior curative intent local therapy, n (%)	81 (32.8)	91 (31.6)	74 (34.7)	70 (32.9)
Prior docetaxel treatment, cycle number	*N* = 241^d^	*N* = 282^d^	*N* = 213	*N* = 213
Median (range)	10 (1–143)	10 (1–83)	10 (1–61)	10 (1–58)
Second line therapy				
Docetaxel, n (%)	34 (13.8)	0	30 (14.1)	0
ARAT,^c^ n (%)	209 (84.6)	284 (98.6)	182 (85.5)	211 (99.1)
Other, n (%)	4 (1.6)	4 (1.4)	1 (0.5)	2 (0.9)
Reason for discontinuing docetaxel, n (%)				
PD	209 (84.6)	233 (80.9)	184 (86.4)	189 (88.7)
AE + other^e^	37 (15.0)	53 (18.4)	29 (13.6)	24 (11.3)
Palliative radiation therapy, n (%)				
Previous treatment	80 (32.4)	88 (30.6)	68 (31.9)	64 (30.1)
Concurrent treatment	8 (3.2)	8 (2.8)	8 (3.8)	5 (2.4)
Initial cabazitaxel dose, n (%)				
≤ 20 mg/m^2^	153 (61.9)	186 (64.6)^f^	134 (62.9)	134 (62.9)^f^
> 20 mg/m^2^	94 (38.1)	102 (35.4)^f^	79 (37.1)	79 (37.1)^f^

*AE* adverse event; *ARAT* androgen receptor axis-targeted agent; *ECOG PS* Eastern Cooperative Oncology Group performance status; *NA* not assessed; *PS* propensity score; *PD* progressive disease; *PSA* prostate-specific antigen; *SD* standard deviation; *TNM* tumour, nodes, metastases

^a^Patient characteristics were collected at the start of cabazitaxel treatment; therefor, baseline characteristics before the third-line treatment is unknown for patients in the 2^nd^ ARAT arm as they received cabazitaxel as forth- or fifth-line treatment

^b^Covariates used were those unlikely to change systematically over treatment lines: age, body surface area, Gleason score, curative intent focal therapy, number of docetaxel treatment cycles, and reason for discontinuation of docetaxel

^c^Abiraterone or enzalutamide

^d^Details on the prior docetaxel treatment was unknown for six patients (*n* = 6)

^e^Details for discontinuing docetaxel treatment was unknown for three patients (*n* = 3) in the CARD-like cohort

^f^Cabazitaxel was received as the forth- or fifth-line therapy

### Time to treatment failure and overall survival

**Fig. 3:**
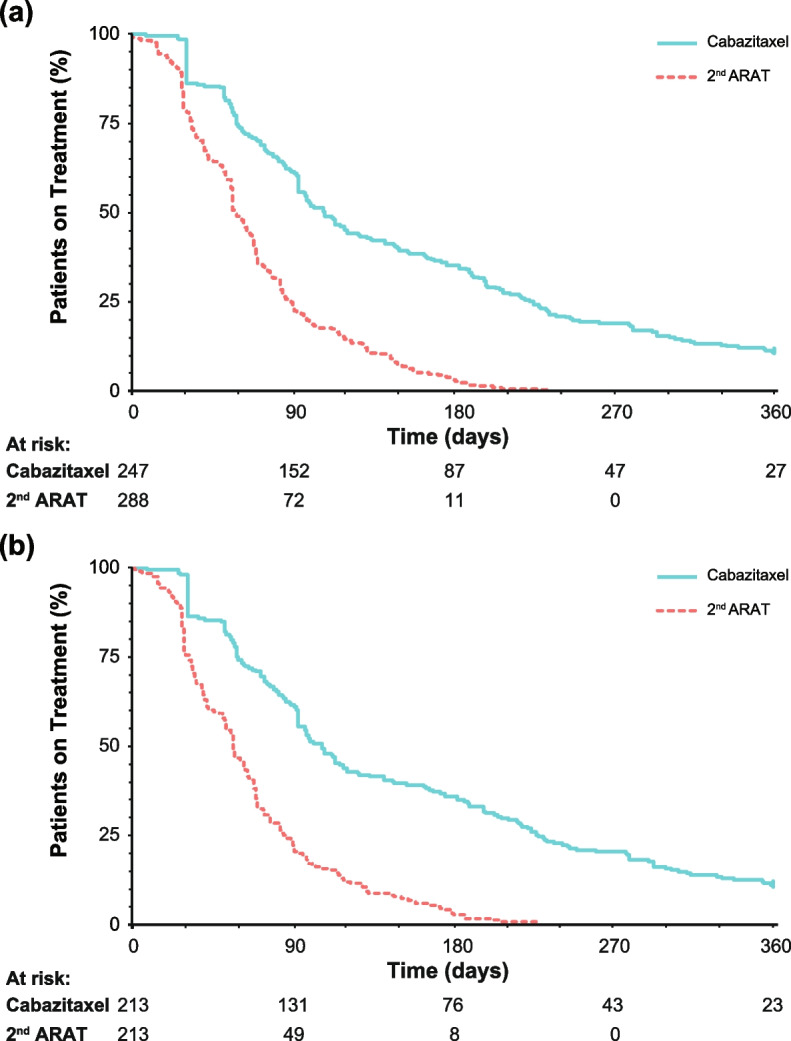
Kaplan–Meier curves for time to treatment failure of; (**a**) third-line treatment (cabazitaxel or the 2^nd^ ARAT) in the unmatched patient cohort, (**b**) the third-line treatment in a PS-matched patient cohort. *ARAT* androgen receptor axis-targeted agent

In the unmatched CARD-like cohort, the median (95% CI) TTF of the first ARAT was similar between the cabazitaxel (94 [85–99] days) and second ARAT (98 [89–100] days) arms (Table 2 and Additional file 2: Fig. S1). TTF results of abiraterone and enzalutamide as first and second ARAT are shown in Fig. S2 (Additional file 2). TTF of the third-line cabazitaxel and second ARAT arms were 109 (94–128) days and 58 (57–66) days, respectively, with a HR (95% CI) of 0.339 (0.279–0.413) favouring cabazitaxel (Fig. 3a and Table 2). Similar results were obtained from the adjusted analysis (Table 2). Likewise, in the PS-matched CARD-like cohort, TTF of third-line cabazitaxel was 108 (94–122) days and that of second ARAT was 57 (53–64) days, with a HR of 0.323 (95% CI 0.258–0.402) favouring cabazitaxel (Fig. 3b and Table 2).

**Table 2:** Time to treatment failure at third-line cabazitaxel or 2^nd^ ARAT

	**Patients, n**		**Event, n (%)**		**Median (95% CI), days**			
	**Cabazitaxel at 3**^**rd**^** line**	**2**^**nd**^** ARAT**^**a**^ **at 3**^**rd**^** line**	**Cabazitaxel** **at 3**^**rd**^** line**	**2**^**nd**^** ARAT**^**a**^ **at 3**^**rd**^** line**	**Cabazitaxel** **at 3**^**rd**^** line**	**2**^**nd**^** ARAT**^**a**^ **at 3**^**rd**^** line**	***P*-value**^**b**^	**HR (95% CI)**^**c**^
First ARAT^a^ at 1^st^ or 2^nd^ line								
Unmatched	247	288	247 (100.0)	288 (100.0)	94 (85–99)	98 (89–100)	NA	NA
3^rd^-line treatment								
Unmatched	247	288	221 (89.5)	288 (100)	109 (94–128)	58 (57–66)	< 0.001	0.339 (0.279–0.413)
PS-matched^d^	213	213	191 (89.7)	213 (100)	108 (94–122)	57 (53–64)	< 0.001	0.323 (0.258–0.402)

*ARAT* androgen receptor axis-targeted agent; *CI* confidence interval; *ECOG PS* Eastern Cooperative Oncology Group performance status; *HR* hazard ratio; *NA* not available; *PS* propensity score; *PSA* prostate-specific antigen; *TNM* tumour, nodes, metastases

^a^Abiraterone or enzalutamide

^b^Log-rank test (two-sided)

^c^Cox proportional hazards model

^d^Covariates were: age, body surface area, Gleason score, curative intent focal therapy, number of docetaxel treatment cycles, and reason for discontinuation of docetaxel

**Fig. 4:**
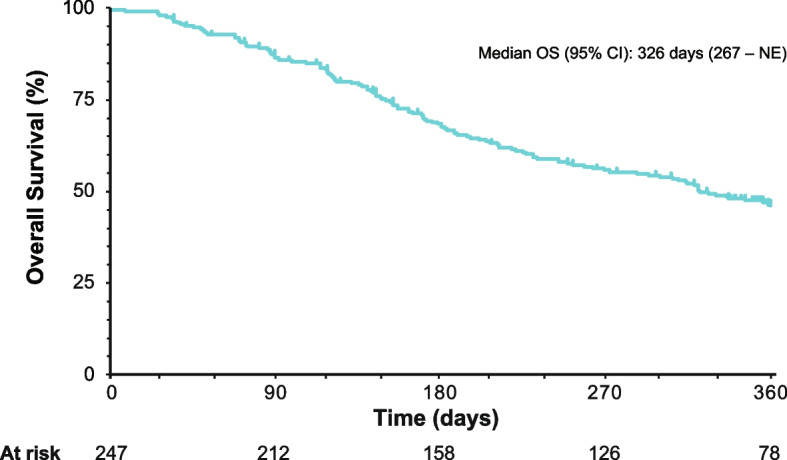
Kaplan–Meier curve for overall survival of patients receiving cabazitaxel at third-line therapy (*N* = 247). *CI* confidence interval; *NE* not estimable; *OS* overall survival

In the unmatched CARD-like cohort, median (95% CI) OS of the cabazitaxel arm was 326 (267– not estimable) days (Fig. 4).

## Discussion

Real-world evidence has been gaining regulatory acceptance as drug discovery, development, and translation evolves [18]. New approaches are enabling collection of better post-marketing data and translation of that data to healthcare practice. Efforts to validate results from clinical trials in the real-world setting, particularly for breakthrough treatments that address serious unmet medical needs, are of paramount importance.

In the CARD clinical study, the frequency of adverse events (AEs) was similar between the cabazitaxel and the second alternative ARAT arms, though AEs leading to treatment discontinuation were reported more frequently with cabazitaxel [16]. Cabazitaxel led to significantly improved imaging-based PFS (8.0 months vs 3.7 months, respectively) and OS (13.6 months vs 11.0 months, respectively; risk of death, HR 0.64, 95% CI 0.46–0.89, *P* = 0.008) compared with the alternative ARAT [16]. Furthermore, patients receiving cabazitaxel had improved quality of life compared with those receiving a second alternative ARAT [19].

In this PMS study, the TTF of the third-line therapy for patients receiving an alternative ARAT was half of that for patients receiving cabazitaxel (58 days vs 109 days, respectively). Whereas imaging-based PFS was assessed as the primary endpoint in the CARD trial, TTF is generally considered to be more appropriate as a measure of effectiveness in clinical practice, because patients may discontinue therapies due to toxicity despite lack of disease progression, in daily practice. The median TTF of cabazitaxel in this study was 109 days and the median PFS in the CARD trial was 4.4 months [16]. There are several possible reasons why the effectiveness of cabazitaxel was worse in this study compared with the CARD trial. First, TTF can be expected to be shorter than PFS because TTF incorporates a wider range of events (e.g. treatment discontinuation due to AEs). In that regard, the frequency of ADRs leading to discontinuation in the cabazitaxel arm of this real-world study was non-negligeable at 14.6% (Additional file 1: Table S1). Second, a lower dose of cabazitaxel (< 25 mg/m^2^) was more frequently used in this study than in the CARD trial (81.4% vs 21.4%, respectively) [16]. Furthermore, the patient cohort in this PMS (both cabazitaxel and second ARAT arms) had more advanced disease than the CARD cohort in terms of Gleason score and serum PSA level. In fact, the OS of the cabazitaxel arm in the real-world was shorter (median, 326 days; 95% CI, 267– not estimable) than that in the prospective CARD trial (median, 413 days; 95% CI, 350–532) [16], which might be attributed to the more advanced disease of the former or lower exposure to cabazitaxel in clinical practice than in the trial. Despite the above differences, the effectiveness of cabazitaxel relative to a second ARAT in this PMS study was in line with that of the CARD trial [16].

The CARD trial enrolled patients who progressed within 1 year on the first ARAT [16]. We emphasize that in this PMS study, only 5 out of 561 patients received the first ARAT for > 1 year. Among those who received the first ARAT for a year or less, only 20% received the therapy for more than 6 months (Additional file 2: Fig. S1) with a median TTF less than 100 days (Table 2). In the CARD trial, 50% of patients received their first ARAT for 6–12 months [16], and another study reported a median PFS of 6.6 months in a patient population receiving their first ARAT following docetaxel [5]. Compared with the patient population of these studies, the patients analysed in this PMS study appears to have been more resistant to ARAT. Nonetheless, TTF of the cabazitaxel arm was longer than that of the second ARAT arm with HR (95% CI) of 0.339 (0.279–0.413) favouring cabazitaxel.

Molecular mechanisms of cross-resistance between ARAT treatments include androgen receptor (AR) splice variants, AR overexpression, AR mutations, and glucocorticoid upregulation [20]. Cell-based analysis has demonstrated that docetaxel confers cross-resistance to cabazitaxel and that this is mediated by increased expression of *ABCB1* [21]. Similar analysis also suggested the existence of intra cross-resistance within ARATs or taxanes, whereas inter cross-resistance between these drug classes does not develop in practice [22]. Prospective randomised clinical trial studies have confirmed cross-resistance between abiraterone and enzalutamide and suggested that abiraterone followed by enzalutamide may provide greater benefit compared with the opposite sequence [8]. In the present analysis, cross-resistance between the first ARAT and the second alternative ARAT was evident, whereas it was unclear which sequencing order was more beneficial in the patient population from this PMS (Additional file 2: Fig. S2a and S2b). Nevertheless, the clinical benefit of cabazitaxel following docetaxel and an ARAT was evident, further supporting the sequential use of an ARAT and cabazitaxel for the treatment of mCRPC in the real-world.

In this analysis, more than half of the CARD-like cohort received sequential ARAT, consistent with recent global real-world studies showing frequent sequential use of ARAT [9, 10]. Our study suggests that patients who progress within 1 year on their first ARAT treatment may be more likely to respond to cabazitaxel than to a second alternative ARAT in a subsequent treatment, confirming the findings of the CARD clinical trial in a real-world Japanese patient population.

### Limitations

Care is needed when interpreting the effectiveness results of this study, which was an observational, non-blinded, non-randomized, non-controlled study design, which lends inherent risks for bias. A surrogate measure of effectiveness (TTF) was used in this study instead of using the same measure of efficacy as the CARD trial (imaging-based PFS). In this *post-hoc* analysis of a Japanese PMS of cabazitaxel, patient characteristics were collected at the start of cabazitaxel treatment; therefore, baseline characteristics *before* the third-line treatment were unknown for patients in the second ARAT arm (because they received cabazitaxel as a fourth- or fifth-line treatment). Whereas the proportions of patients receiving abiraterone and enzalutamide as a second ARAT were balanced in the CARD trial (46.8% and 53.2%, respectively), the majority of the patients in our study received enzalutamide followed by abiraterone (91.3%), which may not have been the optimal sequencing of the two drugs [8]. Finally, the observation period was limited to 1 year.

### Future studies

The study analysed data collected between September 2014 and June 2015. Since then, the prescribing landscape has changed with a trend toward using ARATs in the first line of treatment of mCSPC [6], as well as in combination with docetaxel [23, 24]. Future investigations are needed to see whether progression within 1 year on first ARAT for mCSPC would implicate resistance to the alternative ARAT in patients who then progressed to mCRPC.

## Conclusions

Consistent with the CARD trial, cabazitaxel demonstrated superior effectiveness over a second alternative ARAT in a real-world patient population in Japan, despite the population having more advanced disease status and a lower dose of cabazitaxel being more frequently administered, than in the CARD trial. Cross-resistance between the first ARAT and the second alternative ARAT was evident.

## Supplementary Information

**Additional file 1: Table S1. **Exposure to cabazitaxel, PSA response, and reasons for treatment discontinuation in the CARD-like cohort (cabazitaxel arm). **Table S2.** ADRs in the CARD-like cohort (cabazitaxel arm).

**Additional file 2: Fig. S1.** Kaplan–Meier curves for time to treatment failure of the first ARAT (received as first or second line of treatment). ARAT androgen receptor axis-targeted agent. **Fig. S2.** Kaplan–Meier curves for time to treatment failure of abiraterone and enzalutamide as the first ARAT (a) or second alternative ARAT (b).

## Data Availability

This post-marketing surveillance was conducted under the Japanese Ministerial Ordinance on Good Post-marketing Study Practice for Drugs (GPSP), and due to the characteristics of the surveillance in the regulation, the scope of permission for data sharing is limited to the content described in the paper; however, any inquiries should be directed to the corresponding author.

## Abbreviations

ARAT: Androgen receptor axis-targeted agent
CI: Confidence interval
ECOG PS: Eastern Cooperative Oncology Group performance status
HR: Hazard ratio
mCRPC: Metastatic castration-resistant prostate cancer
mCSPC: Metastatic castration-sensitive prostate cancer
PC: Prostate cancer
PMS: Post-marketing surveillance
PS: Propensity score
PSA: Prostate-specific antigen
SD: Standard deviation
TTF: Time to treatment failure
TNM: Tumour, nodes, metastases

## Footnotes

1 Alternatively referred to as androgen receptor targeting agents (ARTAs).
