# Supplementary material for: Real-world effectiveness of third-line cabazitaxel in patients with metastatic castration-resistant prostate cancer: CARD-like analysis of data from a post-marketing surveillance in Japan
Source: BMC Cancer. 2023 Jun 13;23:538. doi: 10.1186/s12885-023-10998-w (PMC10262372; doi:10.1186/s12885-023-10998-w)
Supplement: Supplementary file 1 — Additional file 1: Table S1. Exposure to cabazitaxel, PSA response, and reasons for treatment discontinuation in the CARD-like cohort (cabazitaxel arm). Table S2. ADRs in the CARD-like cohort (cabazitaxel arm). [file 12885_2023_10998_MOESM1_ESM.docx]

**Table S1** Exposure to cabazitaxel, PSA response, and reasons for treatment discontinuation in the CARD-like cohort (cabazitaxel arm).

|  | Cabazitaxel at 3^rd^ line | | |
| --- | --- | --- | --- |
|  | Overall  (N=247) | Initial cabazitaxel dose | |
|  |  | ≤20 mg/m^2^  (N=153) | >20 mg/m^2^  (N=94) |
| Exposure to cabazitaxel |  |  |  |
| Number of cycles, n | 4.0 (1–17) | 4.0 (1–17) | 4.0 (1–15) |
| TTF,^a^ days | 109 (8–365) | 108 (26–365) | 114 (8–365) |
| Cumulative dose, mg/m^2^ | 80.0 (15.0–385.0) | 80.0 (15.0–385.0) | 85.5 (22.0–375.0) |
| Relative dose intensity, % | 68.6 (20.9–101.0) | 64.6 (20.9–95.0) | 76.6 (36.9–101.0) |
| Baseline PSA, ng/mL | 138.7 (0–16697.2) | 145.5 (0–16697.2) | 135.1 (1.1–3144.7) |
| PSA response,^b^ n (%) | 28 (11.3) | 13 (8.5) | 15 (16.0) |
| Cabazitaxel discontinuation due to ADRs | 36 (14.6) | 21 (13.7) | 15 (16.0) |

Values are median (range) unless indicated otherwise.

^a^Defined as the time from the first day to 30 days after the last day of treatment.

^b^Defined as ≥50% decrease from baseline PSA of ≥5 ng/mL.

*ADR* adverse drug reaction; *PSA* prostate-specific antigen; *TTF* time to treatment failure.

**Table S2** ADRs in the CARD-like cohort (cabazitaxel arm).

|  | Cabazitaxel at 3^rd^ line (N=247) | |
| --- | --- | --- |
|  | Any grade, n (%) | Grade ≥3, n (%) |
| Any ADR | 214 (86.6) | 175 (70.9) |
| Hematologic ADRs |  |  |
| Anemia | 36 (14.6) | 20 (8.1) |
| Leukopenia | 28 (11.3) | 17 (6.9) |
| Febrile neutropenia | 44 (17.8) | 42 (17.0) |
| Neutropenia | 155 (62.8) | 139 (56.3) |
| Thrombocytopenia | 29 (11.7) | 11 (4.5) |
| Bone marrow failure | 1 (0.4) | 1 (0.4) |
| Non-hematologic ADRs |  |  |
| Pneumonia | 7 (2.8) | 5 (2.0) |
| Pyelonephritis | 2 (0.8) | 2 (0.8) |
| Sepsis | 1 (0.4) | 1 (0.4) |
| Septic shock | 2 (0.8) | 2 (0.8) |
| Decreased appetite | 17 (6.9) | 6 (2.4) |
| Peripheral neuropathy | 0 | 0 |
| Interstitial lung disease | 4 (1.6) | 3 (1.2) |
| Pneumonitis | 1 (0.4) | 0 |
| Diarrhea | 29 (11.7) | 10 (4.0) |
| Nausea | 5 (2.0) | 1 (0.4) |
| Vomiting | 3 (1.2) | 2 (0.8) |
| Liver disorders | 8 (3.2) | 3 (1.2) |
| Malaise | 12 (4.9) | 1 (0.4) |
| Pyrexia | 13 (5.3) | 12 (4.9) |

*ADR* adverse drug reaction.
