# Supplementary material for: Real-world effectiveness of third-line cabazitaxel in patients with metastatic castration-resistant prostate cancer: CARD-like analysis of data from a post-marketing surveillance in Japan
Source: BMC Cancer. 2023 Jun 13;23:538. doi: 10.1186/s12885-023-10998-w (PMC10262372; doi:10.1186/s12885-023-10998-w)
Supplement: Supplementary file 2 — Additional file 2: Fig. S1. Kaplan–Meier curves for time to treatment failure of the first ARAT (received as first or second line of treatment). ARAT androgen receptor axis-targeted agent. Fig. S2. Kaplan–Meier curves for time to treatment failure of abiraterone and enzalutamide as the first ARAT (a) or second alternative ARAT (b). [file 12885_2023_10998_MOESM2_ESM.docx]

**Fig. S1** Kaplan–Meier curves for time to treatment failure of the first ARAT (received as first or second line of treatment).
*ARAT* androgen receptor axis-targeted agent.

**
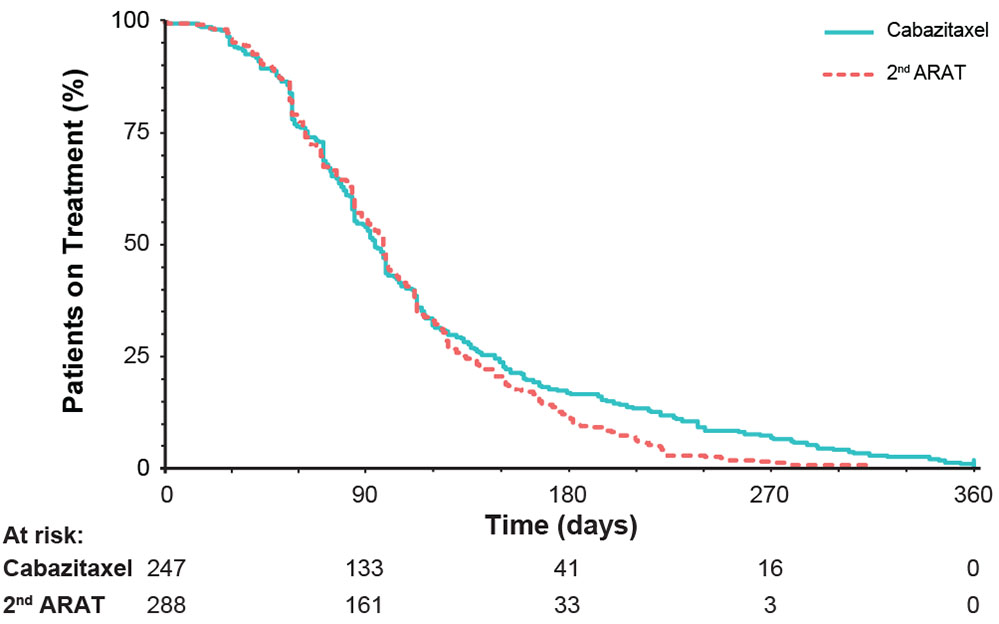
**

**Fig. S2** Kaplan–Meier curves for time to treatment failure of abiraterone and enzalutamide as the first ARAT (a) or second alternative ARAT (b).

**
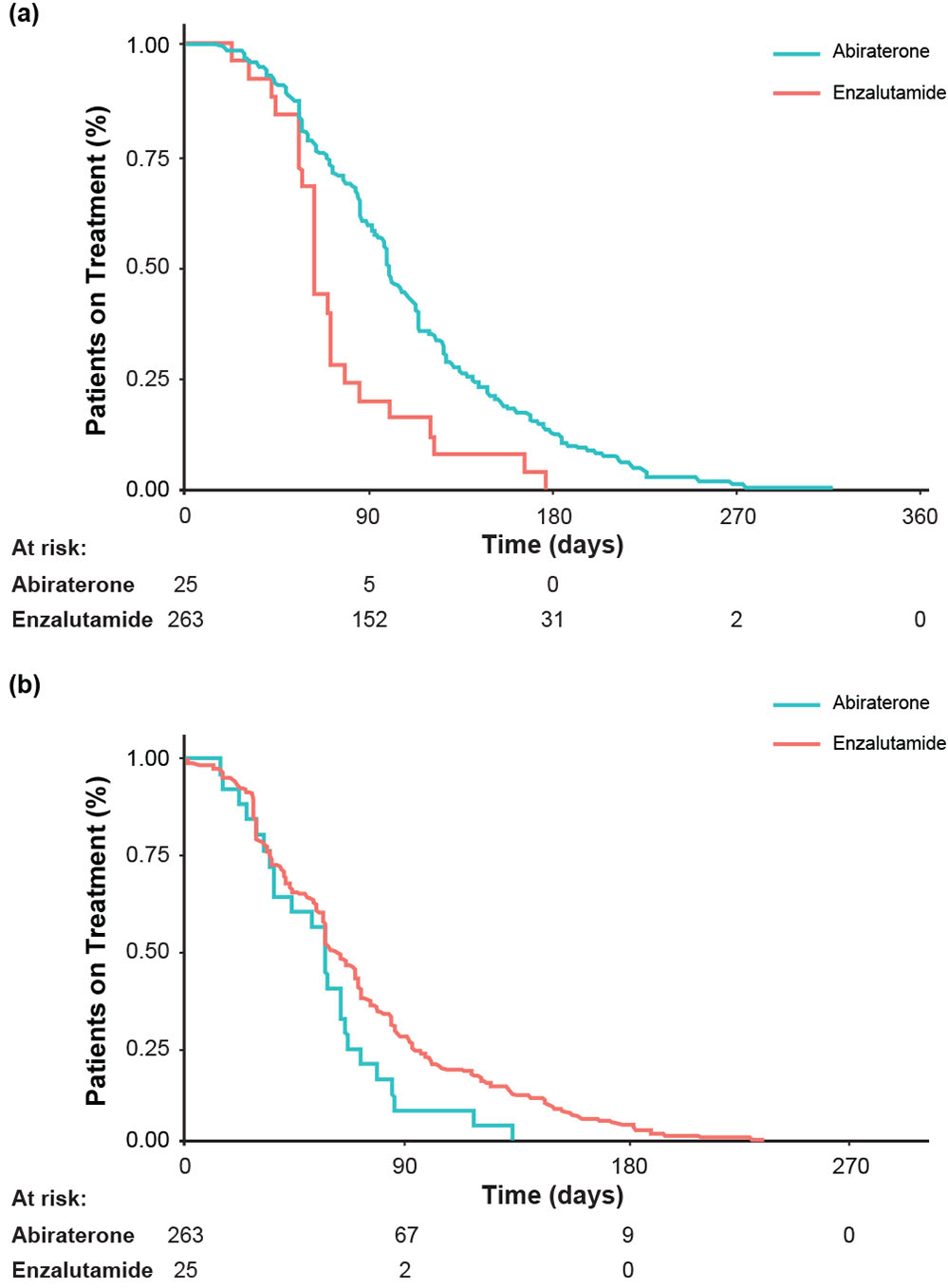
**
